# Nursing in Ireland: The Guinea-Pigs and the Mansion House Cat

**Published:** 1917-04-28

**Authors:** 


### NURSING IN IRELAND: THE GUINEA PIGS AND THE MANSION HOUSE CAT.

FROM AN IRISH CONTRIBUTOR.

Since Miss Cox-Davies and Miss Rundle paid their famous visit to Ireland affairs here have been moving silently but not slowly. As soon as these ladies took their departure the Irish Nurses' Association gathered together such forces as they possess, with the object of circumventing the Royal College of Nursing movement. It was realised that the stagnant waters had been troubled, and that something had to be done, or else peradventure the I.N.A. might die. After much cogitation, therefore, and the button-holing of prominent members of the medical profession, it flashed upon them like an inspiration that a master-stroke would be the granting of diplomas to nurses by the Conjoint Board of Physicians and Surgeons. These worthy dames meet periodically in secret conclave, and between them they decided that these scraps of paper would possess at least a spiritual if not a financial value. Accordingly the proposition was put before the Royal Colleges, not conjointly, but separately, and now behold a great disaster has overtaken it inasmuch as the Royal College of Physicians has rejected it by a substantial majority. This decision is final and decisive, and the result is that the executive of the Irish Nurses' Association have led their followers, if followers they possess, into an Irish bog.

### The Guinea-pigs.

The I.N.A. is an affiliated branch of the Central Powers, or the Central Committee, or whatnot, and is, I venture to suggest, the most autocratic society in Ireland. Although it is run by several English and Scottish ladies it possesses many truly Hibernian characteristics. It is a secret society to begin with. No account of its proceedings is ever permitted to reach its own members, not to mention the general public. Then it is, like most Irish secret societies, a centre of faction. The diploma idea was not concerned with the object of benefiting nurses, but was rather a bomb for the destruction of the Royal College of British Nursing. The Association consists of an executive. A few nurses of the rank and file turn up at the meetings now and then, and if they venture to take a note of the proceedings or to utter a protest they are promptly squelched. And how think you, Sir, is this executive appointed? By ballot? By plebiscite? By referendum? Oh dear, no—but by the simple process of paying a guinea. One might have supposed that during all these years of the existence of the I.N.A. such ardent Registrationists as they love to describe themselves would have compiled a Register of Irish Nurses, and let its members appoint an executive. So far from adopting such a course they framed rules which permit no trained nurse to join the Association unless she is proposed, seconded, and elected unanimously. There are only one or two such exclusive clubs in Ireland. Most of them allow at least one black bean! Their members (consisting mainly, if not entirely, of the staffs of hospitals of which the "executive" are the matrons) are mutes, and their functions and privileges consist of the payment of an annual half-crown in at least one Dublin hospital. This half-crown is deducted from the nurses' salaries. If any of them choose to attend a meeting they may vote provided they amount in number to eight, or a multiple of eight. Every eight votes of ordinary members are equal to one vote by a "guinea-pig." Truly Hibernian, eh? There are no proxies, no votes by country members. Now, these are the ladies who without a blush stood up one after another at the Mansion House meeting to heckle Miss Cox-Davies and Miss Rundle because, forsooth, the College is not founded on democratic principles. Even the Mansion House cat wore a smile on the occasion!

### Will Irish Nurses Discover it?

Irish nurses are bound to find all this out sooner or later, but for their sakes better sooner than later. I am glad to learn that the College is making a promising start. If propaganda work can be commenced at once, all the better. No more worthy example can be followed than that set by Miss Cox-Davies and Miss Rundle. Theirs was the first public meeting of nurses of a general kind ever held here. The nursing profession here is sick of secrecy, and will welcome a policy of publicity as a wholesome change.

